# 

**Published:** 1868-07

**Authors:** 


### CONTENTS.

ORIGINAL COMMUNICATIONS.

Animal Heat ........ 267
Alveolar Abscess ........ 276
Impressions ........ 282
Facts for the People ........ 286

PROCEEDINGS OF SOCIETIES.

Proceedings of the Mississippi Valley Dental Association ........ 288
Illinois State Dental Association ........ 298
Proceedings of the Central Ohio Dental Association ........ 305
Correspondence ........ 307

EDITORIAL.

Explanatory ........ 308
The Examining Board ........ 309
Indiana State Dental Association ........ 309
To the American Dental Association ........ 310

